# Insight into Metal Removal from Peptides that Sequester Copper for Methane Oxidation

**DOI:** 10.1002/chem.201706035

**Published:** 2018-03-05

**Authors:** Arnaud Baslé, Abdelnasser El Ghazouani, Jaeick Lee, Christopher Dennison

**Affiliations:** ^1^ Institute for Cell and Molecular Biosciences Medical School Newcastle University Newcastle upon Tyne NE2 4HH UK

**Keywords:** bioinorganic chemistry, copper, methane, methanotrophs, oxidation

## Abstract

Methanobactins (Mbns) are modified peptides that sequester copper (Cu) methanotrophs use to oxidize methane. Limited structural information is available for this class of natural products, as is an understanding of how cells are able to utilize Mbn‐bound Cu. The crystal structure of *Methylosinus sporium* NR3K Cu^I^–Mbn provides further information about the structural diversity of Mbns and the first insight into their Cu‐release mechanism. Nitrogen ligands from oxazolone and pyrazinediol rings chelate Cu^I^ along with adjacent coordinating sulfurs from thioamides. In vitro solution data are consistent with a Cu^I^–Mbn monomer as found for previously characterized Mbns. In the crystal structure, the N‐terminal region has undergone a conformational change allowing the formation of a Cu^I^_2_–Mbn_2_ dimer with Cu^I^ sites bound by chelating units from adjacent chains. Such a structural alteration will facilitate Cu^I^ release from Mbns.

Methanobactins (Mbns) are a rare example of copper (Cu)‐binding natural products, initially identified in methanotrophic bacteria (methanotrophs).^1^, ^2^, ^3^, ^4^ These organisms require large quantities of Cu to oxidize methane, a reaction of great environmental importance and biotechnological potential,^5^, ^6^, ^7^ using the membrane‐bound particulate methane monooxygenase (pMMO). Mbns are secreted under Cu‐limiting conditions to sequester this metal ion.^1^, ^8^, ^9^ The first Mbn crystallized was that from the model methanotroph *Methylosinus trichosporium* OB3b,^10^ and the structure was subsequently modified and improved (Figure 1 a).^11^, ^12^ The crystal structures of only two other Mbns, both from *Methylocystis* strains, are available,^9^ which exhibit differences to *M. trichosporium* OB3b Mbn (Figure 1). All Mbns characterized to date bind Cu^II^ and Cu^I^, but have a strong preference for the latter with some of the highest known Cu^I^ affinities for biological sites.^9^, ^12^, ^14^, ^15^, ^16^, ^17^, ^18^ This avidity raises the important question of how Mbn‐associated Cu^I^ is released within cells.^9^

**Figure 1:**
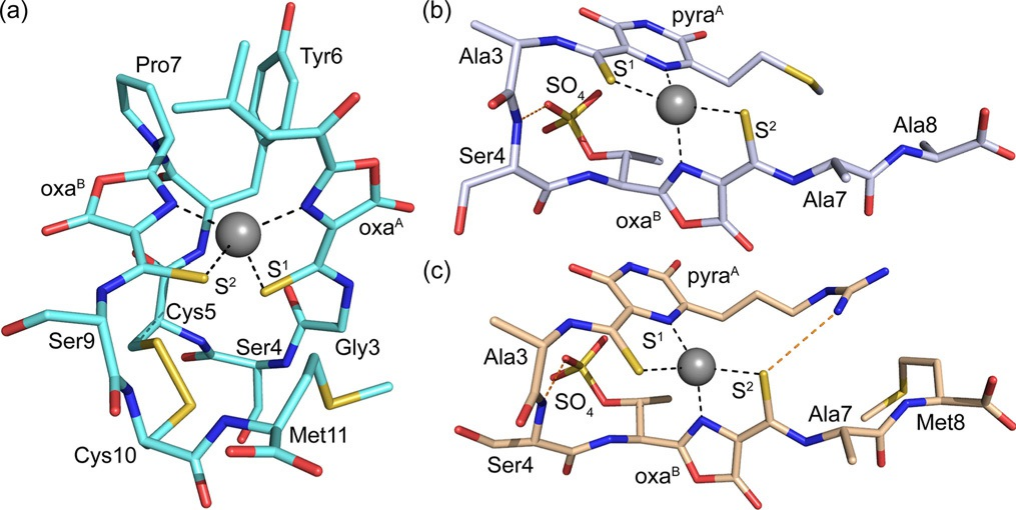
Crystal structures of *M. trichosporium* OB3b (a), *Methylocystis hirsuta* CSC1 (b), and *Methylocystis* strain M (c) Cu^I^–Mbns.^9^, ^12^ Cu^I^ ions are shown as grey spheres and Cu^I^‐ligand and key hydrogen bonds as dashed black and orange lines, respectively. Crystallized *M. hirsuta* CSC1 Cu^I^–Mbn is missing three C‐terminal residues (Thr9, Asn10, and Gly11).^9^, ^13^ Differences between *M. trichosporium* OB3b and *Methylocystis* Mbns include a disulfide bond in the former, a Thr sidechain modified with a sulfate group in the *Methylocystis* Mbns and an N‐terminal pyrazinediol (pyra^A^) ring coordinating in these Mbns in place of the oxazolone (oxa^A^) in *M. trichosporium* OB3b Mbn (oxa^B^ is conserved). The Cu^I^ sites are remarkably similar (see Table S1 in the Supporting Information), regardless of these alterations and the different overall folds of *M. trichosporium* OB3b and the *Methylocystis* Mbns.

The operon that includes the gene (*mbn*A) for the Mbn precursor peptide (leader and core sequences), was initially identified in *M. trichosporium* OB3b, and subsequently in other bacteria, some non‐methanotrophs.^1^, ^2^, ^19^, ^20^ Genes in this Mbn operon are expected to have roles essential to Mbn production and function, as already shown for MbnN^21^ and MbnT,^22^ which are involved in Mbn modification and import, respectively. Bioinformatics of the Mbn operon is limited by the unknown diversity of Mbns. Furthermore, many methanotrophs do not possess the identified Mbn operon, but do have pMMO (present in almost all methanotrophs), including the well‐studied organisms *Methylococcus capsulatus* (Bath), and *Methylomicrobium album* BG8. We attempted to isolate and purify Mbn‐like molecules from media in which these two methanotrophs were grown, but without success (Figure S1 in the Supporting Information). Whether alternative Cu‐sequestering molecules are present in methanotrophs that do not possess the Mbn‐operon remains to be established. The genome of *Methylosinus sporium* NR3K, isolated from Barro Colorado Island, Panama,^23^ is not available, but we have confirmed the presence of the Mbn operon (Figures S2 and S3 in the Supporting Information). MbnA from this methanotroph is similar to that of *M. trichosporium* OB3b, but the crystal structure of the Mbn has unusual features and provides the first insight into how Mbns could release Cu within cells.

The UV/Vis and mass spectra (Figure 2 a–c and Table S2 in the Supporting Information) of the Mbn isolated from *M. sporium* NR3K, as well as its Cu‐binding properties (Figure 2 d–h and Figure S4 and Table S2 in the Supporting Information) are similar to those of previously characterized Mbns, and particularly that from *M. trichosporium* OB3b.^1^, ^9^, ^12^, ^19^, ^24^ The data are consistent with a Cu^I^–Mbn monomer in solution (Figure 1).^9^, ^12^ Furthermore, they indicate a strong preference for Cu^I^ binding (Figure 2 d–h), confirmed by a Cu^I^ affinity of ≈4×10^20^ m ^−1^ (Figure S5 in the Supporting Information). However, the crystal structure of *M. sporium* NR3K Cu^I^–Mbn (Figure 3 a, b) reveals surprising features. The molecule possesses two heterocycles, but the ring at the N‐terminus is a pyrazinediol/pyrazinedione (Figure 3 a and Figure S6 in the Supporting Information). Such a six‐membered ring has only been found to date in *Methylocystis* Mbns (Figure 1 b, c),^9^ with *M. trichosporium* OB3b Mbn, whose sequence is very similar to that of *M. sporium* NR3K Mbn (Figure 3 c), having two five‐membered oxazolones (Figure 1 a).

**Figure 2:**
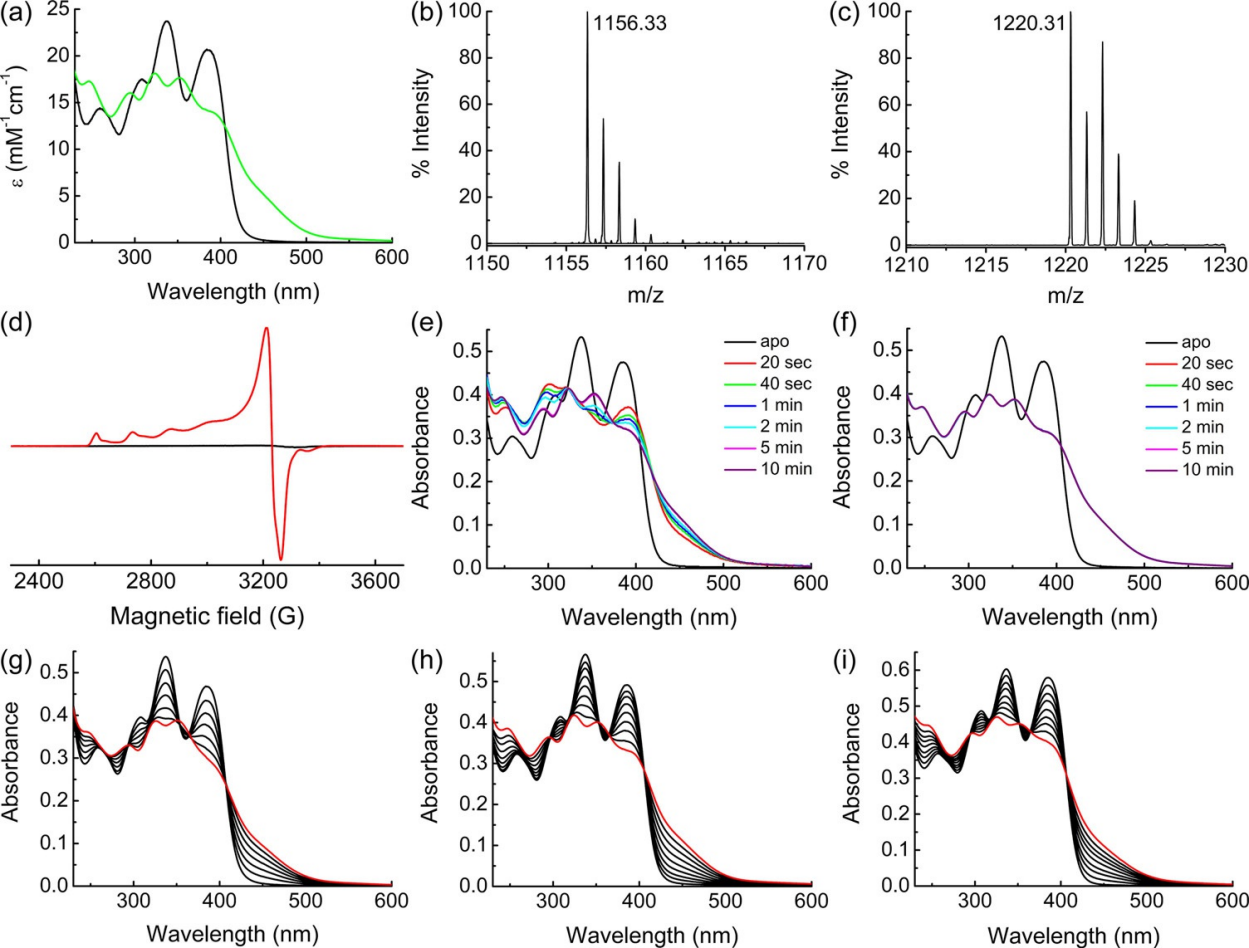
In vitro analysis of *M. sporium* NR3K Mbn. a) UV/Vis spectra of the as‐isolated apo‐ (black line) and Cu‐ (green line) forms. Mass spectra of these are shown in (b, negative ionization mode, [*M*−H]^−^) and (c, positive ionization mode, [*M*+Cu]^+^) respectively. d) Overlay of X‐band EPR spectra (20 K) of as‐isolated Cu–Mbn (2 mm) before (black line) and after (red line) treatment with 10 % nitric acid. The lack of a signal is consistent with the presence of diamagnetic Cu^I^–Mbn, and the addition of nitric acid denatures the Mbn and oxidizes Cu^I^ to Cu^II^. Also shown are UV/Vis spectral changes over time after the addition of ≈1 equivalent of Cu^II^ (e) and Cu^I^ (f) to the apo‐Mbn (22.5 μm). UV/Vis spectral changes upon the titration of up to 1 equivalent of Cu^II^ (g) and Cu^I^ (h) to the apo‐Mbn (22.5 and 24.0 μm respectively) are also included, with the red spectra being the end points of the titrations. The final products in (e) and (g) have UV/Vis spectra the same as that of Cu^I^–Mbn (a, f and h) demonstrating that Cu^II^ is reduced to Cu^I^, as observed for all previously characterized Mbns.^1^, ^9^, ^12^, ^19^, ^24^ The titration of up to 1 equivalent of Cu^I^ is very similar in the form (25 μm) with the disulfide‐bond reduced (i).

**Figure 3:**
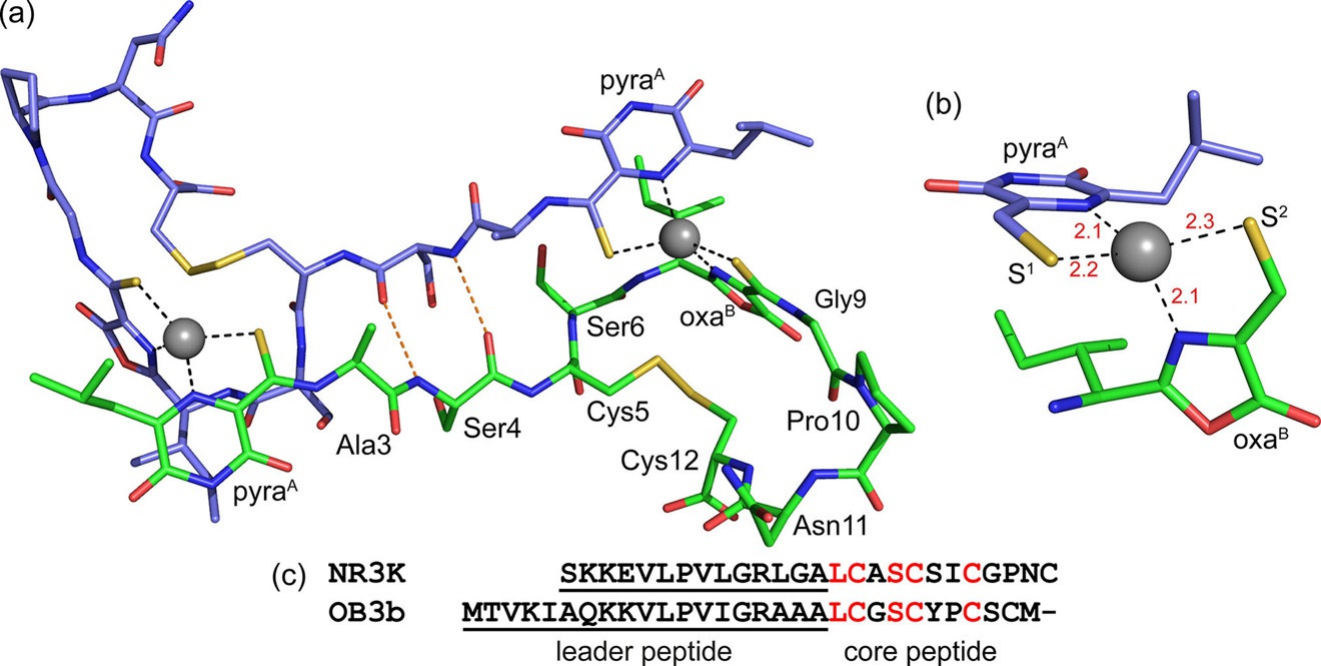
The structure of *M. sporium* NR3K Cu^I^–Mbn. a) The Cu^I^_2_–Mbn_2_ dimer in the crystal structure made from two symmetry‐related monomers that are coloured green and slate, Cu^I^ ions are shown as grey spheres and Cu^I^‐ligand and key hydrogen bonds as dashed black and orange lines, respectively. A close up of one of the Cu^I^ sites is shown in (b) with bond distances (Å) in red. c) Comparison of *M. sporium* NR3K and *M. trichosporium* OB3b MbnA amino‐acid sequences (see Supporting Figure S2 and S3) with conserved residues in the core peptides red.

Most unexpectedly, the Cu^I^–Mbn from *M. sporium* NR3K has formed a Cu^I^_2_–Mbn_2_ dimer in the crystal structure with intermolecular Cu^I^ sites (Figure 3 a, b). This arrangement is very different from the monomers observed in all other Cu^I^–Mbn structures (Figure 1).^9^, ^10^, ^12^ The N‐terminus, containing two of the ligands, has formed an extended, almost linear, polypeptide chain (Figure 3 a), stabilized by hydrogen bonds between the backbone carbonyl and amide groups of Ser4 from adjacent molecules, which mimics a small section of anti‐parallel β‐strand. Adjacent to Ser4 in *M. sporium* NR3K Mbn is Cys5 that forms a disulfide bond with the C‐terminal Cys12 (Figure 3 a, c). The *M. trichosporium* OB3b Mbn also possesses a disulfide bond involving Cys5 (Figure 1 a),^12^ but in this case with the penultimate Cys10 residue (Figure 3 c). Disulfide bond cleavage has a limited influence on *M. sporium* NR3K apo‐Mbn and its Cu^I^‐binding properties (Figure 2 i and Figure S5 and S7 in the Supporting Information), as observed previously for *M. trichosporium* OB3b Mbn,^12^ further highlighting the similarity of these Mbns in solution.

The distorted tetrahedral S_2_N_2_ Cu^I^ sites found in the *M. sporium* NR3K Cu^I^_2_–Mbn_2_ dimer have two coordinating thioamides/enethiols that form chelating units with nitrogen atoms from adjacent heterocyclic rings (Figure 3 a, b). The C‐terminal nitrogen ligand is derived from the oxazolone group, as in all structurally characterized Cu^I^–Mbns (Figure 1),^9^, ^12^ whilst the N‐terminal coordinating nitrogen is provided by the pyrazinediol ring (Figure 3 a, b), as in *Methylocystis* Cu^I^–Mbns (Figure 1 b, c).^9^ There are second‐coordination sphere changes at these intermolecular Cu^I^ sites (Figure 3 a) compared to other Cu^I^–Mbns (Figure 1). Regardless, these Cu^I^ sites (Figure 3 b) have remarkably similar structures to those in other Cu^I^–Mbns (Table S1 in the Supporting Information).^9^, ^12^

All Mbns investigated have very high affinities for Cu^I^ that are in the 10^20^ to 10^21^ m ^−1^ range.^9^, ^12^ The values for *M. sporium* NR3K and *M. trichosporium* OB3b Mbns are alike (see Figure S5 in the Supporting Information), as are their reduction potentials (*E*_m_ values of ≈640 mV for both, see Figure S8 in the Supporting Information). Therefore, their calculated^12^ affinities for Cu^II^ are also similar, albeit ≈8 orders of magnitude weaker than those for Cu^I^. As we have pointed out previously,^9^, ^12^ such high Cu^I^ affinities imply that removing the metal must present a challenge to a cell. Oxidation of Cu^I^–Mbns could facilitate release due to the lower affinities of Mbns for Cu^II^, but the high *E*_m_ values will make conversion to the Cu^II^‐forms difficult in a cell.^9^

The high affinity ligand bathocuproine disulfonate (BCS) is used to measure how tightly Mbns bind Cu^I^ (for example, Figure S5 in the Supporting Information). This bulky 1,10‐phenanthroline derivative with methyl groups adjacent to the two coordinating nitrogen atoms is able to remove Cu^I^ from both *Methylosinus* and *Methylocystis* Mbns,^9^, ^12^ and equilibration is relatively fast (occurring in less than 1 min for *M. sporium* NR3K Mbn). Given the tight Cu^I^ affinities of Mbns, removal by BCS cannot take place via a dissociative mechanism (the unassisted off‐rate for Cu^I^ would be ≈10^−12^ s^−1^), and this large ligand must access the Cu^I^ site, with transfer involving a transient Mbn‐Cu^I^‐BCS intermediate. The Atx1 family of cytosolic Cu^I^ metallochaperones (vide infra) have also been suggested to form transient Atx1–Cu^I^–ligand intermediates through their CXXC Cu(I)‐binding motif with both BCS,^25^ and bicinchoninic acid.^26^ Formation of such intermediates for Cu^I^–Mbns is less straightforward due to them having coordinatively saturated sites (Figure 1). The structure of the Cu^I^_2_–Mbn_2_ dimer provides the first indication of how this can occur. A conformational change at the N‐terminus of *M. sporium* NR3K Cu^I^–Mbn results in the loss of the pyrazinediol and thioamide ligands (Figure 3 a). A structural change such as this could be triggered by a molecule that is able to coordinate Cu^I^; BCS in affinity measurements or another Mbn molecule as seen at the high concentrations used for Cu^I^–Mbn crystallization. Cu^I^ transfer occurs between Mbns in solution (including between *M. trichosporium* OB3b and *Methylocystis* Mbns),^9^ and this must involve Mbn–Cu^I^–Mbn intermediates. Such a species has been stabilized in the crystal structure of the *M. sporium* NR3K Cu^I^_2_–Mbn_2_ dimer. A related mechanism facilitates Cu^I^ transfer from the high affinity,^15^, ^16^, ^18^ but in this case coordinatively unsaturated, sites of Atx1s to the metal‐binding domains of Cu^I^‐transporting P‐type ATPases.^27^, ^28^, ^29^ Although human Atx1 (ATOX1) is a monomer in solution with a two‐coordinate Cu^I^ site bound via its CXXC motif, in the crystal structure it forms a dimer with an intermolecular tetrahedral Cu^I^‐S(Cys)_4_ site.^28^ This structure provided strong support for the suggested ligand‐exchange mechanism of Cu^I^ transfer,^27^ which was later shown to be correct.^29^

The crystal structure of *M. sporium* NR3K Cu^I^–Mbn demonstrates for the first time that this family of molecules can undergo a conformational change with the metal ion bound. This involves the region of the molecule prior to the first Cys residue of its disulfide bond. Most of the core MbnA peptides from *Methylosinus* strains (Figure S3 in the Supporting Information) exhibit sequence similarities that suggest they will release Cu^I^ via a related mechanism. For example, all have a Cys at either position four or five, and also as the penultimate or C‐terminal amino acid (in addition to the two Cys residues that are modified), which probably also form a disulfide bond (Figure 1 a and Figure 3 a).^12^ This could be important for minimising structural changes during Cu^I^ release. Alternatively, although disulfide bond cleavage does not have a very large effect on Cu^I^ affinity (lowers it by ≈2 orders of magnitude), it could assist Cu^I^ release by enabling a larger conformational change if required. A couple of *Methylocystis* Mbns are predicted to have a disulfide bond (Figure S3 in the Supporting Information), but most only have the two Cys residues that provide the sulfur ligands (Figure 1 b, c). Many of the Mbns from these strains also have comparable sequences, and those from *M. hirsuta* CSC1 and *Methylocystis* strain M have similar structures,^9^ with an altered overall fold compared to *M. trichosporium* OB3b Mbn (Figure 1). The conformational change they will undergo to facilitate Cu^I^ release may therefore be different to *Methylosinus* Mbns. The sulfate group on a modified Thr sidechain adjacent to the C‐terminal oxazolone group in *Methylocystis* Mbns (Figure 1 b, c), which can be removed,^9^ may play a role in this process. The presence of an N‐terminal coordinating pyrazinediol ring rather than an oxazolone could influence how Cu^I^ is released from Mbns. However, both provide a heterocyclic nitrogen ligand, are found in *Methylosinus* and *Methylocystis* Mbns and may be more important for recognition with an interacting partner.

Mbns are secreted to sequester Cu and have therefore evolved to bind this metal ion tightly and to not release their cargo extracellularly. However, this makes removing Cu from Mbn within a cell thermodynamically and, considering the structures of monomeric Cu^I^–Mbns (Figure 1), also kinetically unfavorable. In this study we present the crystal structure of a Cu^I^_2_–Mbn_2_ dimer whose formation is only possible due to a conformational change at the N‐terminus. This highlights how Cu^I^ release may be facilitated. Understanding this process is not only important for Cu accumulation in bacteria that secrete Mbns, but also potentially for their development and successful use in the treatment of diseases caused by Cu accumulation.^30^ The next challenge is to identify how conformational‐change‐assisted Cu^I^ release from Mbns occurs within cells.

## Conflict of interest

The authors declare no conflict of interest.

## Supporting information

As a service to our authors and readers, this journal provides supporting information supplied by the authors. Such materials are peer reviewed and may be re‐organized for online delivery, but are not copy‐edited or typeset. Technical support issues arising from supporting information (other than missing files) should be addressed to the authors.

Supplementary
