# Supplementary material for: Insight into Metal Removal from Peptides that Sequester Copper for Methane Oxidation
Source: Chemistry. 2018 Mar 5;24(18):4515–8. doi: 10.1002/chem.201706035 (PMC5947558; doi:10.1002/chem.201706035)
Supplement: Supplementary file 1 — Supplementary [file CHEM-24-4515-s001.pdf]

CHEMISTRY
A European Journal

Supporting Information

Insight into Metal Removal from Peptides that Sequester Copper for Methane Oxidation

Arnaud Baslé+, Abdelnasser El Ghazouani+, Jaeick Lee, and Christopher Dennison*[a]

chem_201706035_sm_miscellaneous_information.pdf

Table of Contents

Materials and Methods 2

Figure S1. The absence of Mbn-like molecules secreted by *M. capsulatus* (Bath) and *M. album* BG8 5

Figure S2. The sequence of part of the *M. sporium* NR3K Mbn operon 6

Figure S3. Multiple sequence alignment of MbnAs from a selection of methanotrophs 7

Figure S4. Determination of the concentration of apo-Mbn 9

Figure S5. The Cu(I) affinity of *M. sporium* NR3K Mbn. 10

Figure S6. The chemical structure of *M. sporium* NR3K apo-Mbn 11

Figure S7. The influence of disulfide bond reduction on the UV-vis spectrum of *M. sporium* NR3K apo-Mbn 12

Figure S8. Electrochemistry of *M. sporium* NR3K Cu-Mbn 13

Figure S9. Partial 16S ribosomal RNA gene sequences 14

Table S1: Comparison of Cu(I) site structures 15

Table S2: Some key properties of *M. sporium* NR3K apo- and Cu(I)-Mbn 16

Table S3: Crystallographic data collection, processing and refinement statistics 17

References 18

Material and Methods

Methanobactin Production and Purification

Methylosinus sporium NR3K (Genbank accession: EF619620)[1] was grown, Mbn recovered from the spent media, purified by reverse phase high performance liquid chromatography (HPLC) and purity verified by analytical HPLC, all using previously described procedures.[2,3] We also tested the well-studied methanotrophic bacteria *Methylococcus capsulatus* (Bath) and *Methylomicrobium album* BG8,[4] whose genomes do not possess the Mbn operon, for secretion of an Mbn-like molecule. The crude extracts isolated from the spent media in which these two organisms were grown have UV-vis spectra (Figure S1a) similar to those reported previously.[5] HPLC purification of these crude extracts does not give rise to any fractions with spectral properties similar to those of characterized Mbns (Figure S1b,c), but are consistent with the presence of flavins, particularly for *M. capsulatus* (Bath).[6]

PCR Amplification of 16S Ribosomal RNA Genes from *M. sporium* NR3K and *M. trichosporium* OB3b

To confirm the identity of *M. sporium* NR3K,[1] a section of its 16S ribosomal RNA gene, and the corresponding region from *Methylosinus trichosporium* OB3b, were amplified by PCR (Taq polymerase, New England Biolabs) with the following primers; 5'-AGAGTTTGATCCTGGCTCAG-3' (forward) and 5'-GTCCGATTAGCTAGTTGGTG-3' (reverse). The forward primer hybridises to the start of the gene, whilst the reverse primer hybridises to positions 201 to 220 and 199 to 218 bp respectively in *M. sporium* NR3K and *M. trichosporium* OB3b. Following an initial denaturation step at 95°C for 30 s, amplification was performed using 30 cycles consisting of 95 °C for 30 s, 55 °C for 30 s and 72 °C for 1 min, with a final extension step at 72 °C for 5 min. The PCR products were extracted from a 2% agarose gel and sequenced using the above reverse primer. The sequenced 16S ribosomal RNA gene region for *M. sporium* NR3K matches that available in the NCBI database with the expected differences (Figure S9) to the corresponding section from *M. trichosporium* OB3b.

Degenerate PCR Amplification of Part of the *M. sporium* NR3K Mbn Operon

Part of the Mbn operon including the *mbnA* gene was amplified from genomic DNA of *M. sporium* NR3K by PCR (Taq polymerase) with the following primers; 5'-ATGGCTATCAAGATC-3' (forward) and 5'-GCGTCGA**B**GCGTCGAA-3' (reverse, degenerate site **B** is G, T or C). The forward primer was designed (based on the genomes of four

methanotrophs) to hybridise to the start of the *mbnA* gene, whilst the reverse primer was designed (based on the genomes of ten methanotrophs) to hybridise to the *mbnB* gene (bases 484 to 500 in *M. trichosporium* OB3b) giving a predicted fragment size of ~700 bp (677 bp for *M. trichosporium* OB3b). Following an initial denaturation step at 95°C for 30 s, amplification was analysed by gradient PCR using 30 cycles consisting of 95 °C for 30 s, 45 to 60 °C for 30 s and 72 °C for 1 min, with a final extension step at 72 °C for 5 min. A fragment of ~ 700 bp was amplified with an annealing temperature of 47.8 °C, extracted from a 1% agarose gel and sequenced using the above primers. The sequence obtained is shown in Figure S2.

In Vitro Characterization of *M. sporium* NR3K Mbn

M. sporium NR3K Mbn was characterized using previously published methods,[2,3] mainly in 20 mM 4-(2-hydroxyethyl) piperazine-1-ethanesulfonic acid (Hepes) pH 7.5 unless stated otherwise. In summary, electrospray ionization mass spectrometry was used to determine molecular weights. UV-vis spectra were acquired on a PerkinElmer λ35 spectrophotometer, using sealed anaerobic quartz cuvettes (Hellma) when necessary. Fluorescence emission spectra were measured on a Cary Eclipse spectrophotometer. Excitation was at 445 nm and emission spectra were recorded over the 450 – 700 nm range with excitation and emission slits of 5 nm. The concentration of copper (Cu) bound to Mbn was measured by atomic absorption spectrometry (AAS) and used to determine molar absorption coefficients (ε values) of Cu(I)-Mbn. The concentration of apo-Mbn was determined from titrations with Cu(I) in the presence of low concentrations of the chromophoric Cu(I) ligand bicinchoninic acid (BCA, see Figure S4). X-band EPR spectra were acquired (20 K) on a Bruker EMX spectrometer equipped with an ESR900 continuous flow cryostat (Oxford Instruments). Cu(I) affinities (K_b values) were determined by competition experiments with the chromophoric Cu(I) ligand bathocuproine disulfonate (BCS) in 20 mM Hepes pH 7.5 plus 200 mM NaCl as described previously.[2] Apo-Mbn was reduced using immobilized tris(2-carboxyethyl)phosphine gel and the presence of free thiols quantified with Ellman's reagent. The direct measurement of the reduction potential (E_m) of as isolated Cu-Mbn (770 μM) was carried out by cyclic voltammetry on a gold working electrode. Measurements were performed in 2-(*N*-morpholino)ethanesulfonic acid (Mes) pH 6.0 and tris(hydroxymethyl)aminomethane (Tris) pH 7.0, both 20 mM plus 90 mM NaCl and typically at a scan rate of 20 mV/s. The influence of scan rate on current was performed in the 2 to 100 mV/sec range, and quoted E_m values are referenced to the normal hydrogen electrode.

Crystallization, Data Collection, Structure Solution and Refinement

Crystals of *M. sporium* NR3K Mbn were obtained using the sitting drop method of vapor diffusion, mixing 0.1 µL of Cu(I)-Mbn (14 mg/mL) in 20 mM Hepes pH 7.5 with 0.1 µL 1.1 M sodium malonate, 0.1 M Hepes pH 7, 0.5% v/v Jeffamine ED-2001 (pH 7) plus 0.5 % w/v n-Octyl-β-D-glucoside (additive screen, Hampton Research). The crystals took months to form and were frozen in 20% glycerol. Diffraction data were collected (100 K) at the ID29 beamline (ESRF, Grenoble, France). Data were processed and integrated with iMOSFLM[7] and scaled using SCALA.[8] The crystal structure was solved by single-wavelength anomalous dispersion based on Cu sites with the SHELX C/D/E suite.[9] The model was completed by iterative cycles of refinement (Refmac)[10] and model-building in COOT.[11] The model was validated with Molprobity,[12] data collection statistics and refinement details are reported in Table S3 and atomic coordinates have been deposited in the Protein Data Bank with the accession code 4oz7.

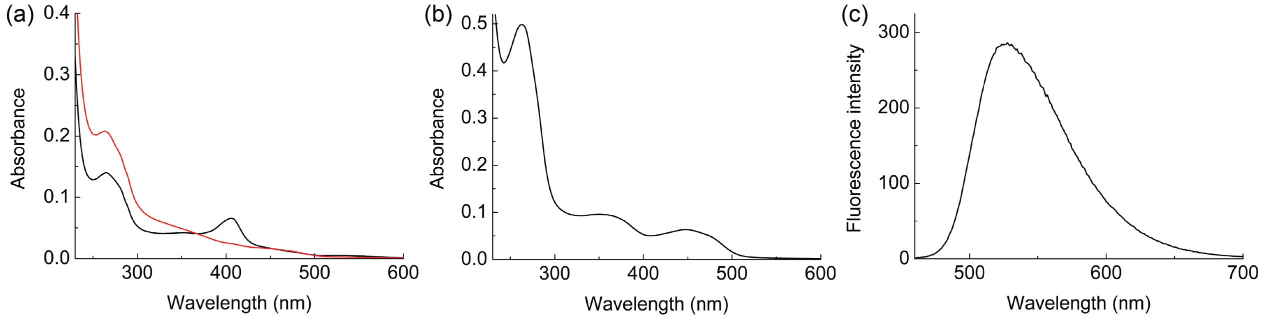

Figure S1. The absence of Mbn-like molecules secreted by *M. capsulatus* (Bath) and *M. album* BG8. a) UV-vis spectra in 10 mM ammonium acetate pH 7 of the crude extracts (eluate from Sep-Pak C_{18} cartridges) from spent media of *M. capsulatus* (Bath, black line) and *M. album* BG8 (red line) cultures. UV-vis (b) and emission (excitation at 445 nm) (c) spectra in 20 mM Hepes pH 7.5 of the main peak obtained after HPLC purification of *M. capsulatus* (Bath) crude extracts. The UV-vis spectrum (b) and the broad emission peak, with a λ_{max} of 528 nm (c), are characteristic of the isoalloxazine ring found in flavins.[6]

AGATCTCCAAGAAGGAAGTGCTCCCGGTTCTCGGCCGCCTCGGCGCGCTGTGCGCCTCGTGCTCGATCTGC
GGCCCGAACTGC**TGA**GTTCGCCGCGCGCCGCCCCGGCGCTTGCGCCGGGGCGGCGCATTTTGCGTGGAATC
ATTCTCGGAGGCGTCTTCGAA**ATG**CAGATCGGCTTCAATTTCACGACAAACCCGACGCTCGATATCGTTCA
GAGGATGATCCGAGAGCGTCAGATCGATTATTGCGAGCTGCTCATCGATAATTTTCTCCACATCCCTCCGC
GGGAGTTGGAGAGCGTTTTCAAATGCCCGCTCGCCTTTCACATCATGTTCTCGAGGTTTCTCGAAAGCGAT
GCCGAATTTCTCGCCAAATTTGCGAAGCGATTGCGGGAGCTGATCGACGCGCTGAATCCGCTCTATGTCTC
GGACCACGTCCTGCGTTTCACGCACGAGGGCCGGCGTCTCTATCATCTCGGAGAGATCGATTACGAAACGG
AATACGACCTCGTCAAGCAACGCGTTTCACGATGGCAGGACATGGTCGGACGTCGCCTGTATCTCGAGAAT
TATCCATCGCTCATGGAAGGCGGATGGGAGGCGCCGGCCTTTTTCGAGCGGCTCACGAAGGAAACCGGCGC
AGGGGTTCTA

Figure S2. The sequence of part of the *M. sporium* NR3K Mbn operon. Reliable sequencing data was obtained ~37 base pairs upstream of the reverse (the final ten 5' base pairs whose sequence is determined by the forward primer did not give good sequencing data) and ~26 bases downstream of the forward primer. Over 83% of the above sequence has been confirmed by sequencing both strands, including the part of the *mbn*A gene coding for the core peptide. The *mbn*A and *mbn*B genes are coloured blue and green respectively with the stop and start codons in bold and underlined.

```
NR3K                 SKKEVLPVLGRLGALCASCS---ICGP--NC
OB3b            MTVKIAQKKVLPVIGRAAALCGSC-----YPC-SCM
3S-1            MTVKIAQKKVLPVIGRAAALCGSC-----YPC-SCM
LW3-1           MAIKIAKKEVLPVVGRLGAMCSSCP---MCGP-LCP
LW3-2           MAINIVKRTTLVVNGRSGADCGTA-----CW----G
LW4             MTIKVVKKEILPVIGRVQAMCACNPP----WC-GTC
PW1             MAIKIAKKEVLPVVGRLGAMCSSCP---MCGP-LCP
R-45379-1       MAIKIAKKEVLPVVGRLGAMCSSCP---MCGP-LCP
R-45379-2       MAINIVKRTTLVVNGRSGADCGTA-----CW----G

CSC1            MTIRIAKRITLNVVGRAGAMCAST-----CAA-TNG
LW5-1           MAIKISKKEVLPVVGRLGAMCSSCP---MCGP-LCP
LW5-2           MAINIVKRTTLVVNGRTGADCGTA-----CW----G
OBBP-1          MAIKIVKKEILPVIGRVQAFCSSCSGGGQCGC-GPA
OBBP-2          MTIKIVKRTALAVNGRAGADCGTA-----CW----A
S285            MAIKIAKRIIMSVIGRAGACCGSH-----CTP--VR
SB2             MTIRIAKRITLNVIGRASARCAST-----CAA-TNG
SC2             MTIRIAKRITLNVIGRAGAMCAST-----CAA-TNG
SV97            MTIRIAKRITLNVIGRASARCAST-----CAA-TNG

                *::.: ::  : * **  * *.
```

Figure S3. Multiple sequence alignment of MbnAs from a selection of methanotrophs produced using T-coffee.[13] Asterisks indicate fully conserved residues with the ':' and '.' symbols highlighting strongly and weakly similar residues respectively. The leader and core peptides are grey and black respectively, and the first five amino acids of *M. sporium* NR3K MbnA are omitted as these are included in the sequence of the forward primer used to amplify the gene. The first nine sequences are for MbnAs from the following *Methylosinus* strains: NR3K - *M. sporium* NR3K (this work); OB3b - *M. trichosporium* OB3b (NCBI reference sequence NZ_ADVE02000001.1, coding DNA sequence (CDS) region 3652950-3653042); 3S-1 - *Methylosinus sp*. 3S-1 (NZ_LXWX01000069.1, 6316-6408); LW3-1 and LW3-2 - *Methylosinus sp*. LW3 (NZ_AZUO01000002.1, 74640-75074 and 1431-1514 respectively); LW4 - *Methylosinus sp*. LW4 (NZ_KB900627.1, 373011-373106); PW1 - *Methylosinus sp*. PW1, (NZ_JQNK01000009.1, 1065071-1065256); R-45379-1 - *Methylosinus sp*. R-45379, (NZ_LUUM01000265.1, 41083-41517); R-45379-2 - *Methylosinus sp*. R-45379 (NZ_LUUM01000262.1, 22986-23069). Also shown are the sequences of MbnA from ten *Methylocystis* strains: CSC1 - *Methylocystis hirsuta* CSC1;[14] LW5-1 and LW5-2 - *Methylocystis sp*. LW5 (NZ_JMKQ01000005.1, 153147-153245 and 254529-255344 respectively); OBBP-1 - *Methylocystis parvus* OBBP (NZ_AJTV01000041.1, 14684-14791); OBBP-2 - *Methylocystis parvus* OBBP (NZ_AJTV01000003.1, 133267-133455); S285 - *Methylocystis bryophila* strain S285 (CP019948.1, 1393877-1393966); SB2 - *Methylocystis sp*. SB2 (NZ_AYNA01000143.1, 19989-20846); SC2 - *Methylocystis sp*. SC2 (HE956757.1, 1456489-1457328); SV97 -

Methylocystis rosea SV97 (NZ_KB889963.1, 344327-344419) The only conserved amino acid in the core peptide is Cys2. The sequences from *M. trichosporium* OB3b and *Methylosinus* 3S-1 are identical, as are those from *Methylosinus* sp. PW1, *Methylocystis* sp. LW3 and *Methylosinus* sp. R-45379, with the MbnA from *Methylocystis* sp. LW5 having a single difference in its sequence to these three MbnAs.

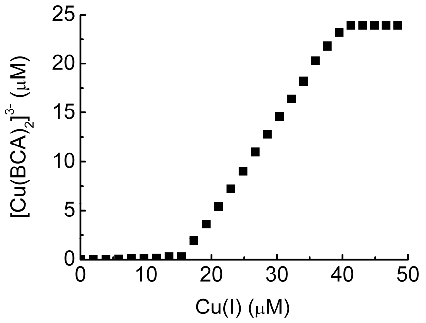

Figure S4. Determination of the concentration of apo-Mbn. Plot showing the formation of $[Cu(BCA)_2]^{3-}$ upon titrating Cu(I) into a mixture of *M. sporium* NR3K apo-Mbn and BCA (50 μM) in 20 mM Hepes pH 7.5. The Cu(I) concentration at which $[Cu(BCA)_2]^{3-}$ starts to form provides the apo-Mbn concentration allowing ε values to be determined.

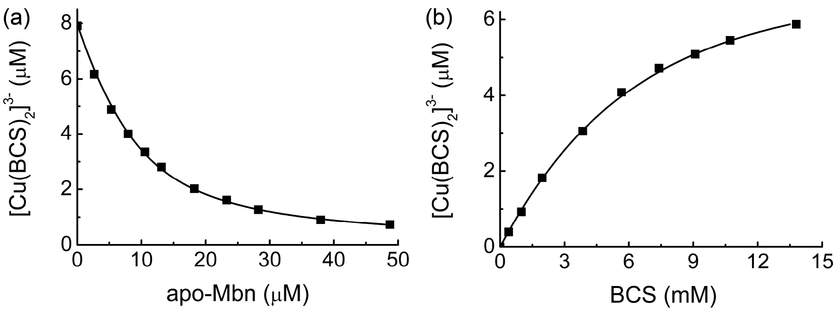

Figure S5. The Cu(I) affinity of *M. sporium* NR3K Mbn. Titrations of apo-Mbn (a) into a mixture of BCS (5 mM) and Cu(I) (7.9 μM), and (b) of BCS into Cu(I)-Mbn (7 μM) in 20 mM Hepes pH 7.5 plus 200 mM NaCl. At each point in the titrations the mixture was left for ~10 min to equilibrate prior to measuring the absorbance at 483 nm from which the $[Cu(BCS)_2]^{3-}$ concentration is determined. The black lines are fits of the data to equation (2) in ref. 2 giving K_b values (Cu(I) affinities) of (3.8 ± 0.2) × 10^{20} M^{-1} and (3.9 ± 0.1) × 10^{20} M^{-1} respectively using an overall stability constant (β_2) for $[Cu(BCS)_2]^{3-}$ of $10^{19.8}$ M^{-2}.[15] This is the β_2 value used previously when determining the Cu(I) affinities of Mbns giving a K_b of (6-7) × 10^{20} M^{-1} for *M. trichosporium* OB3b Mbn.[2] If the revised value[16] of $10^{20.8}$ M^{-2} is used the Cu(I) affinities of the Mbns are an order of magnitude tighter. It should also be pointed out that a number of assumptions are made in the analysis of the above data, primarily due to the absorbance of Cu(I)-Mbn at the wavelength used (483 nm) to quantify $[Cu(BCS)_2]^{3-}$.[2] Therefore the values determined in this way should be taken as very good estimates of the Cu(I) affinities. The lower Cu(I) affinity for *M. sporium* NR3K Mbn than *M. trichosporium* OB3b Mbn is consistent with the result of a competition assay between these, analysed by HPLC.[3] The Cu(II) affinity of *M. sporium* NR3K Mbn is ~ 2 × 10^{12} M^{-1}, calculated using the Cu(I) affinity and the reduction potential (Figure S8).[2] The Cu(I) affinity of *M. sporium* NR3K Mbn decreases by two orders of magnitude when the disulfide bond is reduced, as is also the case for *M. trichosporium* OB3b Mbn.[2]

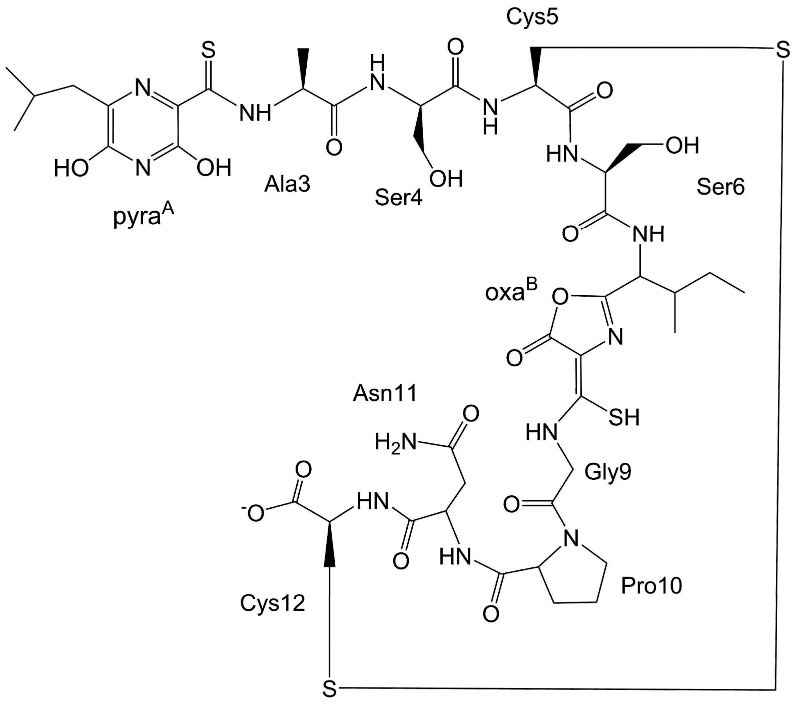

Figure S6. The chemical structure of *M. sporium* NR3K apo-Mbn. The calculated mass 1156.33 Da (as a negative ion, $C_{44}O_{16}N_{13}S_4H_{62}$) matches the experimental mass 1156.33 Da (negative ion mode). The six-membered heterocyclic ring (pyra^{A}) is shown as a pyrazinediol, but could exist as a pyrazinedione or as an intermediate form. A tautomer other than a pyrazinediol would require the non-coordinating nitrogen to be protonated. Careful analysis of our high resolution (0.80 Å) diffraction data[3] shows no indication of protonation at this position in *M. hirsuta* CSC1 Cu(I)-Mbn (2ygi), with some evidence for a small amount in *Methylocystis* strain M Cu(I)-Mbn (2ygj). The resolution of the diffraction data for *M. sporium* NR3K Cu(I)-Mbn (Table S3) is not sufficient to assess this aspect of the six-membered ring structure and we therefore show it in the pyrazinediol form as in the *Methylocystis* Mbn structures. The non-modified amino acid residues are numbered as in the MbnA core peptide.

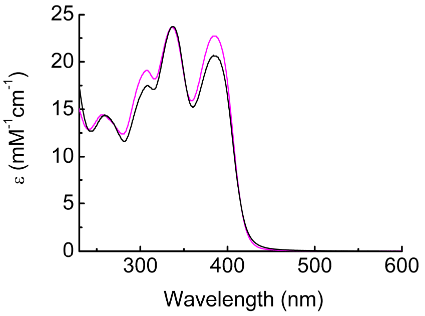

Figure S7. The influence of disulfide bond reduction on the UV-vis spectrum of *M. sporium* NR3K apo-Mbn. UV-vis spectra of apo-Mbn (black line) and of the form in which the disulfide bond has been reduced (magenta line) in 20 mM Hepes pH 7.5.

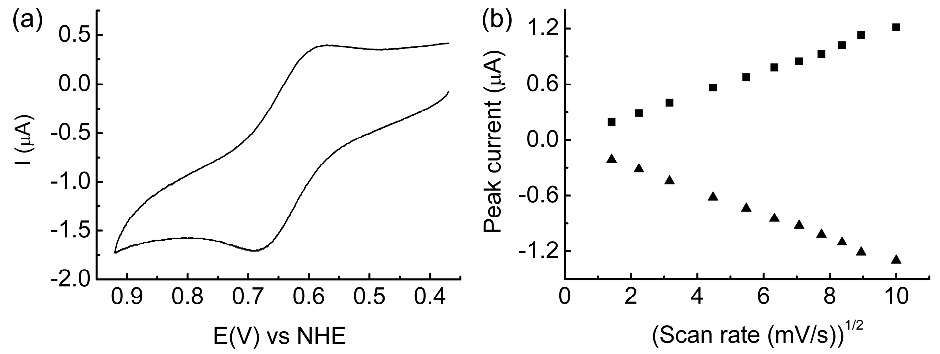

Figure S8. Electrochemistry of *M. sporium* NR3K Cu-Mbn. Cyclic voltammogram at a scan rate of 20 mV/s (a) and scan rate dependence of the cathodic (■) and anodic (▲) peak currents (b) in 20 mM Mes pH 6.0 plus 90 mM NaCl. A quasi-reversible electrochemical response is observed (a) with similar anodic and cathodic peak currents and a peak separation of ~ 80-90 mV, giving an E_m value of 630 ± 5 mV (640 ± 5 mV is obtained from a less ideal response in 20 mM Tris pH 7.0 plus 90 mM NaCl). These E_m values are similar to that of *M. trichosporium* OB3b Cu-Mbn (640 mV at pH 7.5).[2]

```
NR3K            AGAGTTTGATCCTGGCTCAGAACGAACGCTGGCGGCAGGCCTAACACATGCAAGTCGAAC
seq-NR3K        ATAGTTTTATCCTGGCTCAGAACGAACGCTGGCGGCAGGCCTAACACATGCAAGTCGAAC
OB3b            AGAGTTTGATCCTGGCTCAGAACGAACGCTGGCGGCAGGCTTAACACATGCAAGTCGAAC
seq-OB3b        AGAGTTTGATCCTGGCTCAGAACGAACGCTGGCGGCAGGCTTAACACATGCAAGTCGAAC
                * ***** ******************************** *******************

NR3K            GC-TGTAGCAATACAG-AGTGGCAGACGGGTGAGTAACGCGTGGGAACGTGCCTTTCGGT
seq-NR3K        GC-TGTAGCAATACAG-AGTGGCAGACGGGTGAGTAACGCGTGGGAACGTGCCTTTCGGT
OB3b            GGGCGCAGCGATGCGTCAGTGGCAGACGGGTGAGTAACGCGTGGGAACGTACCTTTCGGT
seq-OB3b        GGGCGCAGCGATGCGTCAGTGGCAGACGGGTGAGTAACGCGTGGGAACGTACCTTTCGGT
                ***   * *** ** *   ********************************* *******

NR3K            TCGGAATAACTCAGGGAAACTTGAGCTAATACCGGATACGCCCTTCGGGGGAA
seq-NR3K        TCGGAATAACTCAGGGAAACTTGAGCTAATACCGGATACGCCCTTCGGGGAAA
OB3b            TCGGAATAACTCAGGGAAACTTGAGCTAATACCGGATACGCCCTTAGGGGGAA
seq-OB3b        TCGGAATAACTCAGGGAAACTTGAGCTAATACCGGATACGCCCTTAGGGGAAA
                ********************************************* **** **
```

Figure S9. Partial 16S ribosomal RNA gene sequences. Sections of the 16S ribosomal RNA gene sequences of *M. sporium* NR3K (NR3K, GenBank accession number: EF619620.1) and *M. trichosporium* OB3b (OB3b, NCBI reference sequence: NR_112024.1) are aligned using ClustalW[17] with those obtained for the amplified sections sequenced (seq-NR3K and seq-OB3b respectively). Reliable sequencing data was obtained ~27 base pairs after the reverse primer and the bases highlighted in red and blue are those that differ in *M. sporium* NR3K and *M. trichosporium* OB3b respectively.

Table S1: Comparison of the Cu(I) site structure of *M. sporium* NR3K Cu(I)-Mbn with those of other Cu(I)-Mbns for which crystal structures are available.

Cu-ligand bond distances (Å)	*M. sporium* NR3K (4oz7)[a]	*M. trichosporium* OB3b (2xjh)[b]	*M. hirsuta* CSC1 (2ygi)[c]	*Methylocystis* strain M (2ygj)[d]
Cu–$N^{pyraA/oxaA}$[e]	2.06	2.01	2.10	2.03
Cu–S^1	2.24	2.40	2.25	2.27
Cu–N^{oxaB}	2.16	2.05	2.08	2.03
Cu–S^2	2.29	2.36	2.30	2.27
Angles (deg.)				
$N^{pyraA/oxaA}$–Cu–S^1	87.2	85.6	87.2	87.5
$N^{pyraA/oxaA}$–Cu–N^{oxaB}	114.8	126.8	110.0	122.2
$N^{pyraA/oxaA}$–Cu–S^2	128.9	131.2	125.8	131.2
S^1–Cu–N^{oxaB}	116.2	117.4	119.2	107.9
S^1–Cu–S^2	125.7	110.0	128.8	120.8
N^{oxaB}–Cu–S^2	86.9	87.7	87.8	88.3

[a] Average for the sites between the two molecules in the asymmetric unit and their symmetry-related chains that form dimers. [b] Average for the two molecules in the asymmetric unit.[2] [c] Average for the four molecules in the asymmetric unit.[3] [d] Single molecule in the asymmetric unit.[3] [e] A pyrazinediol ($pyra^A$) ring provides this nitrogen ligand in *M. sporium* NR3K and the *Methylocystis* Cu(I)-Mbns, whilst both nitrogen ligands are provided by oxazolone rings (oxa) in *M. trichosporium* OB3b Cu(I)-Mbn.

Table S2: Some key properties of *M. sporium* NR3K apo- and Cu(I)-Mbn.

		Apo-Mbn	Cu(I)-Mbn
HPLC elution times (min)	Semi-prep. column	26.2	33.7
	Analytical column	11.7	18.6
Molecular weight (Da)		1156.33[a]	1220.31[b]
ε (mM^{-1} cm^{-1})		23.7 (± 0.2)[c]	15.9 (± 0.2)[e]
		20.7 (± 0.5)[d]	

[a], [b] Correspond to the mass of $[M\text{-}H]^-$ and $[M\text{+}Cu]^+$ respectively. [c], [d] ε values (n = 3) at 337 nm and 384 nm respectively for apo-Mbn obtained using concentrations determined from Cu(I) titrations into a mixture of apo-Mbn plus 50 μM BCA (Figure S4). [e] ε value at 296 nm for Cu(I)-Mbn obtained using AAS to quantify Cu (n = 5).

Table S3: Crystallographic data collection, processing and refinement statistics.

	M. sporium NR3K
**Data collection**[a]	
Beamline	ESRF ID29
Date	21/11/2009
Wavelength (Å)	0.98
Resolution (Å)	28.16 – 1.65 (1.74 - 1.65)
Space group	I 2 2 2
Unit-cell parameters: *a*, *b*, *c*	36.72, 39.42, 40.24
$\alpha = \beta = \gamma$ (°)	90
Unit-cell volume (Å^3)	58247
No. of measured reflections	21959 (2133)
No. of independent reflections	3677 (490)
Completeness (%)	99.0 (94.1)
Redundancy	6.0 (4.4)
R_{merge} (%)	8.7 (34.2)
<I>/<σ(I)>	11.3 (3.4)
**Refinement statistics**	
R_{work} / R_{free}[b] (%)	22.08 / 25.07
Average B factors (Å^2)	
Protein	23
Solvent	39
Cu	20
No. of non-H atoms	181
RMS deviation from ideal	
Bond angle (°)	2.86
Bond length (Å)	0.012
Ramachandran plot,[c] residues in most favoured regions (%)	100

[a] Values in parenthesis are for the highest resolution shell. [b] 5% of the randomly selected reflections excluded from refinement. [c] Calculated using Molprobity.[12]

References

[1] W. Knapp, D. A. Fowle, E. Kulczycki, J. A. Roberts, D. W. Graham, *Proc. Natl. Acad. Sci. USA* **2007**, *104*, 12040–12045.

[2] A. El Ghazouani, A. Baslé, S. J. Firbank, C. W. Knapp, J. Gray, D. W. Graham, C. Dennison, *Inorg. Chem.* **2011**, *50*, 1378–1391.

[3] A. El Ghazouani, A. Baslé, J. Gray, D. W. Graham, S. J. Firbank, C. Dennison, *Proc. Natl. Acad. Sci. USA* **2012**, *109*, 8400–8404.

[4] R. S. Hanson, T. E. Hanson, *Microbiol. Rev.* **1996**, *60*, 439–471.

[5] D. W. Choi, N. L. Bandow, M. T. McEllistrem, J. D. Semrau, W. E. Antholine, S. C. Hartsel, W. Gallagher, J. Z. Corbin, N. L. Pohl, J. A. Zahn, A. A. DiSpirito, *J. Inorg. Biochem.* **2010**, *104*:1240–1247.

[6] R. Balasubramanian, B. T. Levinson, A. C. Rosenzweig, *Appl. Environ. Microbiol.* **2010** *76*, 7356–7358.

[7] A. G. W. Leslie, H. R. Powell, Evolving methods for macromolecular crystallography. **2007**, *245*, 41-51 ISBN 978-1-4020-6314-5.

[8] P. R. Evans, *Acta Cryst.* **2006**, *D62*, 72–82.

[9] G. M. Sheldrick, T. R. Schneider, *Methods Enzymol.* **1997**, *277*, 319–343.

[10] A. A. Vagin, R. A. Steiner, A. A. Lebedev, L. Potterton, S. McNicholas, F. Long, G. N. Murshudov, *Acta Cryst.* **2004**, *D60*, 2284–2195.

[11] P. Emsley, B. Lohkamp, W. G. Scott, K. Cowtan, *Acta Cryst.* **2010**, *D66*, 486–501.

[12] V. B. Chen, W. B. Arendall, J. J. Headd, D. A. Keedy, R. M. Immormino, G. J. Kapral, L. W. Murray, J. S. Richardson, D. C. Richardson, *Acta Cryst.* **2010**, *D66*, 12–21.

[13] C. Notredame, D. G. Higgins, J. Heringa, *J. Mol. Biol.* **2000**, *302*, 205–217.

[14] L. M. K. Dassama, G. E. Kenney, S. Y. Ro, E. L. Zielazinski, A. C. Rosenzweig, *Proc. Natl. Acad. Sci. USA* **2016**, *113*, 13027–13032.

[15] Z. Xiao, F. Loughlin, G. N. George, G. J. Howlett, A. G. Wedd, *J. Am. Chem. Soc.* **2004**, *126*, 3081–3090.

[16] P. Bagchi, M. T. Morgan, J. Basca, C. J. Fahrni, *J. Am. Chem. Soc.* **2013**, *135*, 18549–18559.

[17] M. A. Larkin, G. Blackshields, N. P. Brown, R. Chenna, P. A. McGettigan, H. McWilliam, F. Valentin, I. M. Wallace, A. Wilm, R. Lopez, J. D. Thompson, T. J. Gibson, D. G. Higgins, *Bioinformatics* **2007**, *23,* 2947–2948.
